# Plasmid-DNA Encoded *gata3* and *eomes* Increase Antibody Response and Performance of an Inactivated Salmonid Alphavirus Subtype 3 Vaccine

**DOI:** 10.3390/pharmaceutics18081033

**Published:** 2026-08-20

**Authors:** Filipe Figueiredo, Kristian Gillebo Sørmo, Aleksander Pettersen, Heng Chi, Jaya Kumari Swain, Toni Erkinharju, Trilochan Swain, Shripathi Bhat, Kim Præbel, Stefano Peruzzi, Jacques Godfroid, Øystein Evensen, Hetron Mweemba Munang’andu, Roy Ambli Dalmo

**Affiliations:** 1 Norwegian College of Fishery Science, Faculty of Biosciences, Fisheries and Economics, UiT-The Arctic University of Norway, N-9019 Tromsø, Norway; filipe.figueiredo@uit.no (F.F.); kristians@arnarlax.is (K.G.S.); aleksander.pettersen@outlook.com (A.P.); chiheng@ouc.edu.cn (H.C.); jaya.k.swain@uit.no (J.K.S.); tjay1975@gmail.com (T.S.); shripathi.bhat@uit.no (S.B.); kim.praebel@uit.no (K.P.); 2 Norwegian Veterinary Institute, N-1431 Ås, Norway; toni.erkinharju@vetinst.no; 3 Department of Arctic and Marine Biology, Faculty of Biosciences, Fisheries and Economics, UiT–The Arctic University of Norway, N-9019 Tromsø, Norway; stefano.peruzzi@uit.no (S.P.); jacques.godfroid@uit.no (J.G.); 4 Department of Paraclinical Sciences, Faculty of Veterinary Medicine, Norwegian University of Life Sciences, N-1431 Ås, Norway; oystein.evensen@nmbu.no; 5 Faculty of Biosciences and Aquaculture, Nord University, N-8026 Bodø, Norway; hetron.m.munangandu@nord.no

**Keywords:** Salmonid alphavirus 3, Atlantic salmon, vaccination, molecular adjuvants, Gata-3, eomes, fish growth, antibody response, histopathology

## Abstract

**Background:** Pancreas disease, caused by salmonid alphavirus subtype 3 (SAV3), remains a significant threat to Atlantic salmon (*Salmo salar* L.) aquaculture in Norway despite the widespread use of inactivated vaccines. Molecular adjuvants represent a promising strategy to improve vaccine efficacy by enhancing immune responses. **Methods:** In this study, we evaluated the immunological and protective effects of two transcription factors, *gata3* and *eomes* encoded by plasmid DNA adjuvants in combination with a low-dose inactivated SAV3 vaccine. Atlantic salmon were immunized with inactivated virus alone or in combination with either *gata3*- or *eomes*-encoding plasmids, followed by SAV3 challenge. Vaccine performance was assessed by growth, antibody response (ELISA and virus neutralization assay), and histopathological scoring of heart and pancreas. **Results:** The effects of *gata3* and *eomes* were context-dependent: *eomes* reduced pancreas lesions at 4 weeks post-challenge, while both plasmid-adjuvant groups showed improved growth at 10 weeks post-challenge compared to the plasmid control. Although antibody responses were increased in all vaccinated groups at 6 weeks post-immunization, the plasmid control performed comparably to the GATA3 or EOMES groups, and no significant differences were detected in the virus neutralization test. Interestingly, the empty plasmids given to control fish exhibited immunostimulatory activity, warranting further investigation. In vitro and in vivo fluorescence microscopy confirmed expression of the transcription factors in transfected cells and at the injection site. **Conclusions:** Our results suggest that transcription factor-encoding plasmids can provide selective adjuvant effects, while also highlighting the potential that plasmid DNA can have as adjuvants in fish vaccine development.

## 1. Introduction

Vaccines are widely used in aquaculture, particularly in Norwegian salmon farming, with an estimated 600 million doses administered annually [1], highlighting the success of vaccination as a prophylactic method. Despite the variety of commercially available vaccines, infectious diseases, especially diseases caused by viruses, still occur frequently [2]. Four main types of vaccines are used in commercial aquaculture globally: inactivated, live-attenuated, subunit, and DNA vaccines [3]. Inactivated whole-organism vaccines are widely utilized for the prevention of bacterial diseases, whereas live-attenuated pathogen vaccines are specifically engineered to retain immunogenicity while eliminating virulence but are less commonly employed. A few DNA vaccines have demonstrated high vaccine efficacy and involve the intramuscular injection of a plasmid vector carrying the gene of interest, leading to antigen expression and immune response [4]. Most vaccines used in Norwegian salmon aquaculture are inactivated, with a notable exception being a DNA vaccine approved for pancreas disease (PD) in 2018 [5]. The Norwegian Atlantic salmon (*Salmo salar*) industry frequently contends with viral diseases that result in significant economic losses and raise concerns about fish welfare. Pancreas disease, caused by salmonid alphavirus (SAV), is one such viral disease. Salmonid alphavirus is a single-stranded, positive-sense RNA virus from the *Togaviridae* family, with six known genotypes (SAV1 through SAV6) that infect Atlantic salmon [6]. Two genotypes, SAV2 and SAV3, are endemic to Norway [7]. While PD outbreaks have decreased in recent years, the disease remains a challenge for the Norwegian aquaculture industry due to its negative economic impact [8]. Consequently, continued improvement and refinement of PD vaccines is still necessary to prevent disease occurrence.

Adjuvants are crucial for enhancing and modulating vaccine immunogenicity [9], particularly in cases where antigens, such as those in inactivated vaccines, fail to elicit a robust immune response [10]. Although high dosages and/or repeated administration of vaccines can improve efficacy [3], this approach is often cost-prohibitive, especially when producing high quantities of inactivated virus. Cost-effectiveness always remains a key consideration in any vaccine development for aquaculture [11]. Additionally, many vaccines currently available for Atlantic salmon in Norway are multivalent [12], and there is a growing trend towards micro-dose application, underscoring the importance of adjuvants in developing effective and long-lasting immune responses.

Adjuvants in commercial vaccines for fish are broadly characterized as oil-based and non-oil-based, with oil-based adjuvants creating a depot effect that continuously releases antigens, thereby enhancing antibody production and protection against pathogens [13]. Despite being the most widely used type of adjuvant, oil-based formulations can have several adverse effects that are detrimental to fish welfare and growth [14,15].

Research into adjuvant signatures and modes of action contributed to the development of an expanded two-signal model that provides a conceptual framework for understanding how adjuvants can modulate distinct stages of the immune response [16,17]. This framework identifies innate activation, antigen presentation, co-stimulatory signals, T helper cell (Th) polarization, and homing signal as potential targets for adjuvant-mediated modulation [18]. Consequently, vaccine adjuvants can enhance immune responses through several, often overlapping mechanisms, including the formation of antigen depots that promote sustained antigen release, activation of pattern recognition receptors (PRRs) and their downstream signaling pathways, induction of local inflammation and immune cell recruitment, enhancement of antigen uptake and presentation, and targeted activation of specific immune pathways [19,20]. By acting at these different stages, adjuvants can shape the resulting immune response toward the desired immune response. In this context, molecular adjuvants comprise immunomodulatory molecules like cytokines, chemokines, transcription factors, and other immune signaling components that can be used to direct or enhance specific components of the immune response [21,22]. An underexplored approach is to encode such molecular adjuvants within DNA vectors, allowing their expression alongside the vaccine antigen and potentially increasing vaccine efficacy by promoting more robust or directed immune responses [23,24,25,26].

Duration of immunity is a critical aspect of vaccine efficacy. Therefore, developing adjuvants that potentially enhance T and B cell activity may be advantageous, especially against intracellular pathogens such as viruses. In this study, two vector-based molecular adjuvants [27], GATA-binding protein 3 (*gata3*) and eomesodermin (*eomes*), were evaluated in combination with an inactivated salmonid alphavirus vaccine in Atlantic salmon. *Gata3* is a transcription factor that regulates the expression of T helper 2 (Th2) cytokine genes such as il4, il5, and il13, which are important for the development of humoral immune responses [28,29]. *Eomes* drives the differentiation and activation of CD8^+^ T cells in mice, boosting their cytotoxic functions by promoting the expression of cytolytic molecules like perforin and granzyme B [30]. Although transcription factors typically regulate gene expression by entering the nucleus [31], recent findings indicate that they can also bind RNA, potentially affecting gene expression [32]. Regardless of the mechanisms by which transcription factors regulate gene expression, our previous studies have shown that *gata3*-encoding plasmids, when injected intramuscularly in Atlantic salmon, express *gata3* at the injection site [33]. Although *eomes*-encoding plasmids have not been tested in vivo, ectopic expression and silencing of *eomes* in primary spleen leukocytes have been shown to regulate IFNγ and granzyme A production [34]. This study aimed to evaluate two transcription factors as molecular adjuvants to enhance the immunogenicity and protective efficacy of a low-dose inactivated virus vaccine.

The current study found that byadding *gata3*- or *eomes*-encoding plasmids to an inactivated SAV vaccine, a strong adjuvant effect was achieved, which improved the vaccine efficacy compared to inactivated PD vaccine.

## 2. Materials and Methods

### 2.1. Experimental Fish and Setup

A single batch of Atlantic salmon eggs (AquaGen AS, Kyrksæterøra, Norway), hatched at the Tromsø Aquaculture Research Station (Tromsø, Norway), were reared until they reached the parr stage (~45 g). The 970 parr to be used were tagged with a passive integrated transponder (PIT-tag; Biomark, ID, USA), weighed, measured, and set at a water temperature of ca. 4 °C and 12/12 h photoperiod (day/night), to trigger smoltification. One week prior to vaccination, temperature was gradually increased at a rate of 0.5 °C per day, until it reached 10 °C. Routine analysis (PCR-based/histopathology) showed that the fish were free from infection by piscine myocarditis virus (PCMV), piscine orthoreovirus (PRV), infectious pancreas necrosis virus (IPNV), pancreas disease virus (PDV/SAV), infectious anemia virus (ISAV), salmon gill poxvirus (SGPV), avirulent infectious salmon anemia, *Branchiomonas cysticola*, and *Candidatus clavochlamydia salmonis* (causing epitheliocystis disease).

The experiment was divided into two periods: immunization and SAV3 challenge (cf. experimental design; graphical abstract). All experimental procedures were carried out following the Norwegian Animal Welfare Act and regulations on experiments with animals (regulation 2017-04-05, no. 451). The trial was approved by the Norwegian Food Safety Authority (approval no: ID11949).

### 2.2. Plasmid Construction Used for Intramuscular Injection

For the construction of the plasmid encoding *gata3* and *eomes*, the open-reading frames of Atlantic salmon *eomes* (EU418014.1) and *gata3* (EU418015) were retrieved from NCBI GenBank and cloned as previously described (Supplementary Table S1) [33,35,36]. From the spleen cDNA library, the genes were sub-cloned into a pTagRFP-N vector (Evrogen, Moscow, Russia) by PCR, using primer sequences with aid of restriction enzymes PstI and XhoI (Promega, WI, USA). All the PCR products of *eomes* and *gata3* genes and pTagRFP-N vector were digested with XhoI and PstI endonucleases (Promega, WI, USA.) and ligated with a T4 DNA ligase (New England Biolabs, MA, USA) to generate the constructs. A re-ligated pTagRFP-N plasmid without the insert was also constructed as a negative control—this also contains, as the other plasmid constructs, the gene encoding RFP. All plasmids were transformed and grown in One Shot TOPO 10 *Escherichia coli* (Invitrogen, CA, USA), isolated using Plasmid Mini Kit (Qiagen, Venlo, The Netherlands), verified by restriction map analysis and DNA sequencing. Purification of plasmids was done using the Qiagen Plasmid Mega or Qiagen Plasmid Giga kits, according to the manufacturer’s protocol. The anion-exchange chromatography purified plasmid DNA was further diluted in phosphate buffered saline, PBS (pH 7.4) (Sigma-Aldrich, Miss, USA) before injection into fish. The concentration of plasmid DNA was determined using a Nanodrop (Thermo Fisher Scientific, MA, USA).

### 2.3. Cell Culture and Transfection Assay

To confirm that the expression vectors were transcribed, the expressions of *gata3* and *eomes* were assessed using Chinook salmon (*Oncorhynchus tshawytscha*) embryonic cells (CHSE-214). CHSE-214 is a continuous epithelial cell line initiated from embryonic tissue of Chinook salmon in 1964 and has been widely used in virology and aquatic animal health [37,38]. Cells were seeded in a standard flask (Nunc, Thermo Fisher Scientific) with L-15 medium (Invitrogen) containing penicillin (60 μg mL^−1^), streptomycin (100 μg mL^−1^), 1% non-essential amino acids (NEAA, Gibco, NY, USA) and 8% fetal bovine serum (FBS) (Sigma-Aldrich, Miss, USA). The CHSE-214 cells were incubated at 20 °C for 1 week. Cells were then washed twice with 10 mL PBS before adding 1.5 mL trypsin (Sigma-Aldrich, Miss, USA). Loosened cells were re-suspended in L-15 medium (8% FBS, 1% NEAA, without antibiotics) and counted in Nucleocounter YC-100 (Chemometec, Allerød, Denmark). Transfections of plasmids were performed using the Invitrogen Neon Transfection System according to the manufacturer’s protocol (Invitrogen). The cells were either transfected with pTagRFP-*eomes*, pTagRFP-*gata3* or pTagRFP-N expressing plasmids in separate wells (1 × 10^5^ cells per well). Mock-transfected cells were considered as control. After 12 h of incubation in the transfection medium, the cells were gently washed and received L-15 medium to maintain cell viability and support optimal growth. After 48 h, the cells were fixed with 4% formaldehyde (*w*/*v*) (Thermo Fisher Scientific) for 30 min, and then DAPI (Sigma-Aldrich) was used for nucleic acid staining according to the manufacturer’s protocol. Micrographs were obtained by inverted fluorescence microscope equipped with DAPI-365 and Texas Red 530-585 filters (Zeiss Axiovert 40 CFL, Zeiss, Jena, Germany).

### 2.4. Immunization

The inactivated virus (IV-LD) vaccine was prepared at NMBU [39]. SAV3 was inactivated by formalin treatment and emulsified in an oil/water suspension by Ultra-Turrax homogenization (Fisher Scientific, MA, USA) using 7.4 parts Montanide ISA763A VG (Seppic, La Garenne-Colombes, France) and 2.6 parts water.

All fish participating in the trial were starved for one day before vaccination. On the vaccination day, the fish (average weight: 44.30 g) were sedated in a 0.02% solution of benzocaine. The oil-adjuvanted vaccine (low-dose inactivated virus) was injected intraperitoneally, while plasmid DNA (pDNA) containing either transcription factor was injected intramuscularly. Vaccine groups, vaccine type, and doses are shown in Supplementary Table S2. During this phase of the trial, fish were sampled at 6- and 10-weeks post-immunization (WPI). At the end of the immunization trial, fish health was inspected by a veterinarian and confirmed to be satisfactory. No mortality or signs of distress were observed during handling, sedation, or vaccination. In addition, during the 10-week immunization phase and subsequent experimental challenge, fish were monitored daily by experienced staff, and no mortality or abnormal clinical signs were detected. If any major clinical side effects had occurred, we were expected to terminate the experiment (following Norwegian and EU rule regarding experimental use of animals).

### 2.5. Salmonid Alphavirus Subtype 3 Challenge

For the viral challenge to take place, 720 fish were transferred to the Fish Health Laboratory at the Tromsø Aquaculture Station, divided across four tanks, and acclimatized for three days prior to challenge. The fish were randomly placed in the four tanks, in a common-garden setup where the same number of fish in each treatment group were kept together. The fish were tagged with a passive integrated transponder (PIT). Salmonid alphavirus subtype 3 was cultured in CHH-1 cells (Sigma-Aldrich) and diluted in phosphate buffer saline (PBS) to a titer of 1 × 10^9^ TCID_50_. On the day of the challenge, all fish were anesthetized in a 0.02% solution of benzocaine and injected intraperitonially with 1 × 10^9^ TCID_50_ in 100 µL PBS. The average fish weight at challenge was 148.8 ± 1.7 g. After challenge, fish were kept in 10 °C seawater with a 24/0 (light/dark) photoperiod. Technical staff at the station performed daily care of the fish. No mortality was observed during the challenge period (up to 10 weeks). Sampling took place at 1, 4, 6, and 10 weeks post-challenge (WPC).

### 2.6. Sampling

At each timepoint for either phase of the trial, 10 fish from each group were killed by benzocaine overdose (Benzoak Vet.; 0.02%/L) (ACD Pharmaceuticals, Leknes, Norway), length and weight were recorded. Blood was withdrawn from the caudal vein into vacutainer tubes containing heparin. The vacutainer tubes were kept in an ice bath. The blood samples were transferred to Eppendorf tubes and centrifuged at 3000 rpm for 10 min, and the collected serum stored at −20 °C. Heart and pancreas were excised and stored in a solution of 4% formalin for later histology processing. Formalin was replaced with 70% ethanol after 24 h of fixation at room temperature.

### 2.7. Specific Growth Rate

Specific growth rate (SGR) was calculated for the individually tagged fish sampled at different timepoints, by using the following formula:

SGR (% day^−1^) = ((ln*W*_2_ − ln*W*_1_)/∆*t*) × 100

where

• *W*_1_ = initial weight (g),
• *W*_2_ = final weight (g),
• Δ*t* = time interval in days,
• ln *=* natural logarithm.

### 2.8. Histology/Histopathology

To prepare the tissues for histopathology, samples fixed in 10% formalin were dehydrated (Citadel 2000, Thermo Fisher Scientific, MA, USA), cleared (Histoclear, National Diagnostics Inc., NC, USA), and embedded in paraffin wax (Histowax, Histolab Products AB, Gothenburg, Sweden) in a Leica EG 1150H (Leica Biosystems, Nusslock, Germany). After embedding, 5 μm slices were cut with a Leica RM2235 microtome (Leica Biosystems, Nussloch, Germany) and stained with hematoxylin and eosin (H&E).

Before H&E staining, the tissue sections were incubated at 60 °C for two hours and washed with Histoclear to remove the paraffin wax. Staining was done in a Leica ST4020 Small Linear Stainer (Leica Biosystems, Nussloch, Germany) using hematoxylin and eosin (Thermo Fisher Scientific, MA, USA), prepared according to the manufacturer’s instructions. After staining, coverslips were mounted using Eukitt (Sigma-Aldrich, Miss, USA) for storage at room temperature and further analyses.

Histological sections were analyzed via a Leica CTR6000 light microscope (Leica Biosystems, Nussloch, Germany), with pictures being taken with a Moticam 5 MP camera (MoticEurope, Barcelona, Spain). Histopathological assessment of the heart and pancreas was carried out to detect pathological changes due to SAV3 infection. Different degrees of degeneration and inflammation of the tissue were used as an indicator (histoscore) for the potential protective effect of a vaccine. This scoring system was developed by the Veterinary Institute in Trondheim, having been modified from McLoughlin et al. [40]. Samples were blinded and scored (1 to 3) by a trained pathologist based on the semi-quantitative scoring system (Table 1).

### 2.9. Enzyme-Linked Immunosorbent Assay (ELISA)

ELISA was performed to measure the antigen-specific antibody response in pre- and post-challenge fish (10 WPI and 6 WPC). In a pre-trial, it was established that the 1:100 dilution of the primary antibody gave optimal results. The dilution of serum samples was, in the pre-run trial, 1:10 to 1:25,600, where dilutions from 1:20 to 1:160 (1:20, 1:40, 1:80, and 1:160) were selected for the main ELISA runs. In summary, rabbit anti-SAV3-E2 [41] was added to each well of the 96-well microtiter plates and left overnight (4 °C) before a 1:2 dilution of SAV3 (1 × 10^3^ TCID_50_) in 1% blocking buffer was added. The plates were incubated for 2 h at room temperature. Serum samples in 1% blocking buffer were then added and left at room temperature for 2 h. Mouse anti-salmon IgG (Sigma-Aldrich, Miss, USA) (was added (100 µL of 1:50 dilution in 1% blocking buffer) to the wells and the plates were left at room temperature for 1 h. Then, 100 µL of horse radish peroxidase (HRP)-conjugated with goat anti-mouse IgG, diluted 1:5000 in 1% blocking buffer, was added to each well and incubated for 1 h at room temperature. Subsequently, 50 µL of o-phenylenediamine (OPD) dissolved in 0.05 M citric acid buffer (pH 5.0) was added. The plates were incubated at room temperature for 30 min, after which 0.05 M HCl (20 µL) was added to stop the enzymatic reaction. All samples were analyzed in triplicate.

### 2.10. Virus Neutralization Assay (VNA)

While an ELISA detects the presence and quantity of specific antibodies in a sample, VNA measures the ability of the serum to block viral infection in cells, thus providing an indirect measure of protective immunity. This protective immunity was tested before and after challenge.

Serum samples (n = 10) from all vaccine and control groups were diluted in Leibovitz’s L-15 Medium (Thermo Fisher Scientific, in triplicate, from 1:10 to 1:1280. Then 50 μL of SAV-3 virus (1 × 10^5^ TCID50) was added to all the wells and mixed with serum. The plates were incubated for 2 h at RT. The wells containing the CHH-1 cells were emptied from growth medium and 100 μL of above-mentioned serum mixed with virus was transferred to the microtiter plates. The plates were incubated and monitored for 7 days at 15 °C. By microscopic examination of the cells, the cells were graded either positive or negative dependent on the cytopathic effect.

### 2.11. Viral Load

Approximately 10 mg of heart tissue was used to extract RNA with a Blood and Tissue RNeasy kit (Qiagen, Venlo, The Netherlands). The manufacturer’s protocol was slightly modified as follows: The tissue was added to an Eppendorf tube (Eppendorf, Hamburg, Germany) containing one steel bead and buffer RLT (600 μL). Homogenization was performed in a TissueLyzer II (Qiagen, The Netherlands) for two cycles of 2 min at maximum speed. The resulting supernatant was transferred to fresh tubes, to which of 500 μL of nuclease-free water and 10 μL of proteinase K (Qiagen, The Netherlands) were added, followed by incubation at 55 °C for 10 min. The remaining steps were carried out according to the manufacturer’s instructions. RNA concentration was determined using a Qubit fluorometer (Thermo Fisher Scientific, MA, USA) with the RNA Broad Range Assay kit. Samples were stored at −80 °C until further processing.

Assessment of viral load in the heart at 4WPC was carried out through RT-qPCR using the method developed by [42], with a Ct value cut off of 37. Quantification was done relative to treatment-specific baseline Ct values obtained at 10 WPI. For each treatment group, the mean Ct value at 10WPI was used as the baseline. ΔCt values were calculated as the difference between the Ct value of each individual fish at 4WPC and the mean baseline Ct value of its corresponding treatment group. Relative viral load was then computed using the $2^{-\Delta \Delta Ct}$ method [43].

### 2.12. Statistical Analysis

All statistical analyses and data visualization were performed in R version 4.4.0 [44]. Normality of continuous variables (e.g., weight, SGR, ELISA absorbance, virus neutralization assay dilutions, histoscore values, and viral load) was assessed using the Shapiro–Wilk test within each timepoint and treatment group. For variables satisfying normality assumptions, homogeneity of variances was evaluated using the median-centered Levene test. Weight and SGR were analyzed using one-way ANOVA within each timepoint when assumptions of normality and homogeneity of variances were met, followed by Tukey’s honestly significant difference (HSD) test for post hoc multiple comparisons. When the assumption of homogeneity of variances was violated (SGR at 10WPI), Welch’s one-way ANOVA followed by Games–Howell post hoc comparisons were used. Variables that did not satisfy the assumptions for parametric analysis (ELISA absorbance, virus neutralization assay dilutions, histoscore values, and viral load) were analyzed using the Kruskal–Wallis test, followed by Dunn’s multiple comparison test with Benjamini–Hochberg (BH) correction for *p-values*. For ELISA absorbance data, differences between timepoints within each treatment group were additionally evaluated using the Wilcoxon rank-sum test with Benjamini–Hochberg correction for *p-values*. Overall differences in virus neutralization assay dilutions between timepoints were evaluated using the Wilcoxon rank-sum test.

In all cases, *adjusted p-values < 0.05* were considered statistically significant. Group means are presented with standard error of the mean (SEM). For viral load data, results are presented as medians with interquartile ranges (boxplots). Statistical comparisons were visualized using *ggbarplot* from the ggpubr [45] package, with brackets and *adjusted p-values* added using *stat_pvalue_manual*. Viral load data were visualized with *geom_boxplot* and *geom_jitter* from the ggplot2 package to show both treatment distributions and individual variation.

Further R packages used were readxl [46] for reading Excel files, tidyverse [47] for data wrangling/plotting, rstatix [48] and effectsize [49] for statistical analysis methods, scales [50] for axis scaling and number formatting, MoMAColors [51] for plot colors, and patchwork [52] for combining plots. Effect sizes to compare SGR across treatments were calculated using Cohen’s d. Values of 0.2, 0.5, and 0.8 correspond to small, medium, and large effects, respectively [53].

## 3. Results

### 3.1. Growth

**Figure 1:**
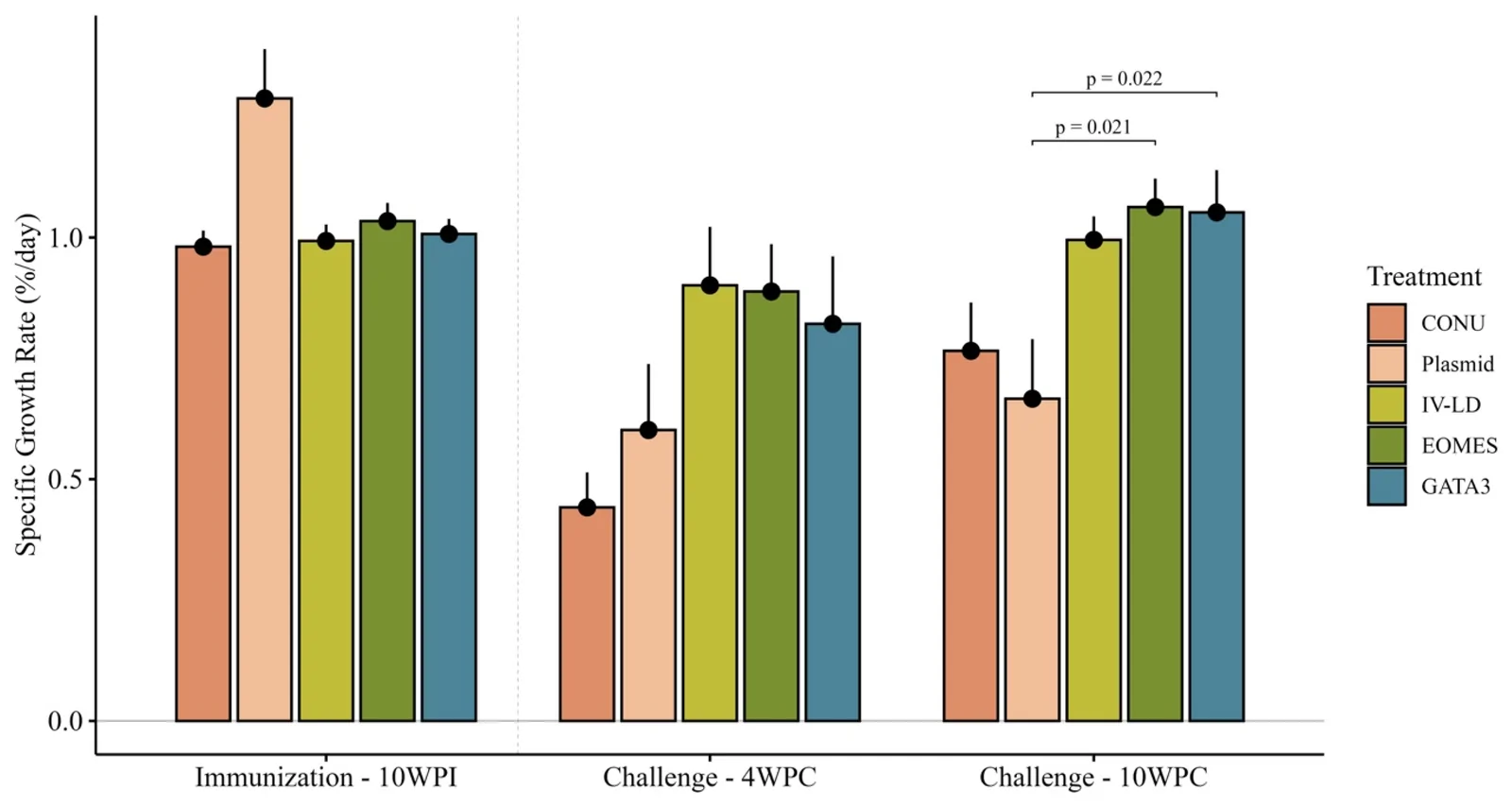
Growth performance of Atlantic salmon assessed in three periods: immunization to 10 weeks post-immunization (10 WPI), challenge to 4 weeks post-challenge (4 WPC), and challenge to 10 weeks post-challenge (10 WPC). Fish were assigned to five treatment groups: CONU (saline control), Plasmid (non-coding plasmid), IV-LD (low-dose inactivated Salmonid Alphavirus subtype 3, SAV3), EOMES (IV-LD + *eomes* transcription factor-encoding plasmid), GATA3 (IV-LD + *gata3* transcription factor-encoding plasmid). Bars represent the specific growth rate (% of weight increase per day). Statistical differences between treatment groups were evaluated using either *Welch’s one-way ANOVA* (10WPI) or a *one-way ANOVA* followed by *Tukey’s HSD* post hoc test (4WPC and 10WPC) with *Benjamini–Hochberg* correction for multiple comparisons. *Adjusted p-values* are shown above statistically significant pairwise comparisons.

Specific growth rate (Figure 1) was used to assess the effects of vaccination on growth performance during the immunization and challenge phases. During the immunization phase (Figure 1: Immunization-10WPI), the Plasmid group had a higher mean SGR than the remaining treatment groups. However, because homogeneity of variances was violated at this timepoint, the treatment effects were assessed using *Welch’s one-way ANOVA*, which detected no significant differences between treatments (*p = 0.125*). The higher mean SGR likely reflects the 20-day delay in immunization caused by vaccine production constraints rather than a treatment effect.

At the start of the challenge (zero weeks post-challenge [0WPC]), a *one-way ANOVA* found no significant differences in mean body weight among groups (*p* = 0.75; mean body weight = 148.8 ± 1.7 g), indicating that fish entered the challenge at comparable sizes. During challenge, significant treatment effects on SGR were detected at both 4WPC (*one-way ANOVA*, *p =* 0.028) and 10WPC (*one-way ANOVA*, *p =* 0.005). Although no pairwise comparisons remained significant following *Tukey’s HSD* correction at 4WPC, comparisons between the CONU and the IV-LD, EOMES, and GATA3 groups exhibited large effect sizes, as determined by *Cohen’s d* (*d =* 1.08–1.64; Supplementary Table S3). At 10 WPC, fish in the Plasmid group had significantly lower SGR compared to the EOMES and GATA3 groups (*Tukey’s HSD, adjusted p-value* = 0.021 and 0.022, respectively). These comparisons were also associated with large effect sizes (*d =* 1.18–1.37; Supplementary Table S3).

At the end of the experiment (10 WPC), the final mean weights (± SEM) were as follows: 279.1 ± 23.9 g for CONU, 235.2 ± 20.3 g for Plasmid, 286.6 ± 15.9 g for IV-LD, 320.6 ± 17.6 g for EOMES, and 321.9 ± 15.5 g for GATA3. Fish in the EOMES and GATA3 groups were significantly heavier than those in the Plasmid group (*Tukey’s HSD, adjusted p-value* = 0.025 and 0.018, respectively) at the end of the experiment.

### 3.2. Transcription Factor Expression

**Figure 2:**
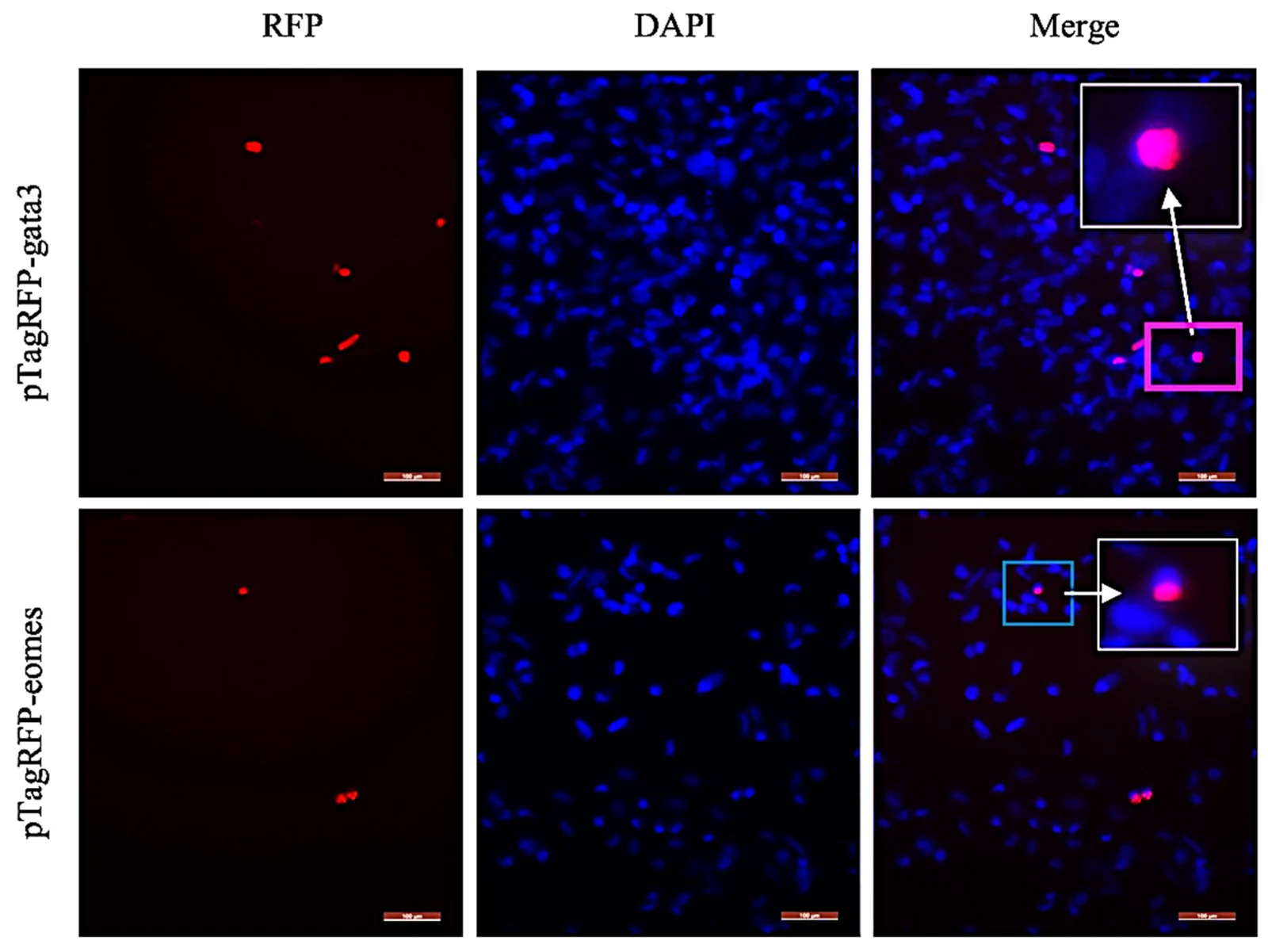
Fluorescence microscopy of CHSE-214 cells transfected with pTagRFP-gata3 (**top row**) and pTagRFP-eomes (**bottom row**). Images were acquired 48 h post-transfection and display: red fluorescent protein (RFP) signal indicating transgene expression (**left**), DAPI staining of cell nuclei (**middle**), and merged images (**right**) showing co-localization of RFP and DAPI. Red fluorescence was localized within the nuclei. Insets in the merged panels highlight examples of nuclear localization (arrows). Blue boxes delineate nuclei of interest. Scale bars = 100 μm.

The in vitro expression of *gata3* and *eomes* in CHSE-214 cells was confirmed via fluorescence microscopy (Figure 2). Detection of the red fluorescent protein (RFP) signal indicates successful plasmid transcription of the genes and expression of the transgenes. The co-localization of RFP with DAPI nuclear staining further confirms that the target transcription factors are being expressed within the cell nuclei (Figure 2).

**Figure 3:**
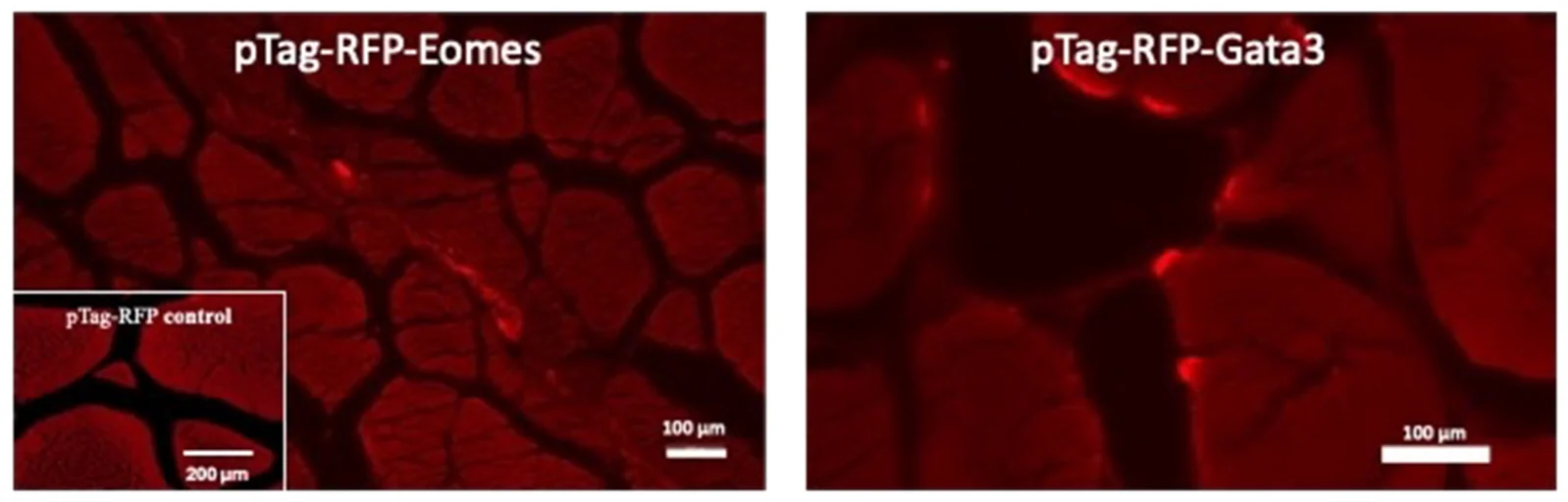
Fluorescence micrographs of muscle tissue sections showing red fluorescence protein (RFP) expression 48 h post-injection with plasmids encoding either *eomes* (**Left image**) or *gata3* (**Right image**). Fluorescence was predominantly localized to structures consistent with the endomysium, the connective tissue enveloping individual muscle fibers, suggestive of expression in resident fibroblasts. Images were captured at 40× magnification. Scale bars represent 100 μm. No specific RFP signal was detected in control muscle sections (inserted in the left image), confirming the specificity of transgene expression.

To confirm in vivo expression of *gata3* and *eomes*, muscle tissue sections from the injection site were prepared without H&E staining to visualize RFP signal, thereby indicating transgene expression. Analysis of these tissue sections revealed *gata3*- and *eomes*-associated RFP expression at the injection site only (Figure 3). Control specimens did not show any specific fluorescence.

In contrast, fluorescence in vaccine injected fish was identified in endomysial cells involved in the production of fascial connective tissue, as well as in other cells associated with the fascial layer.

### 3.3. Antibody Production

**Figure 4:**
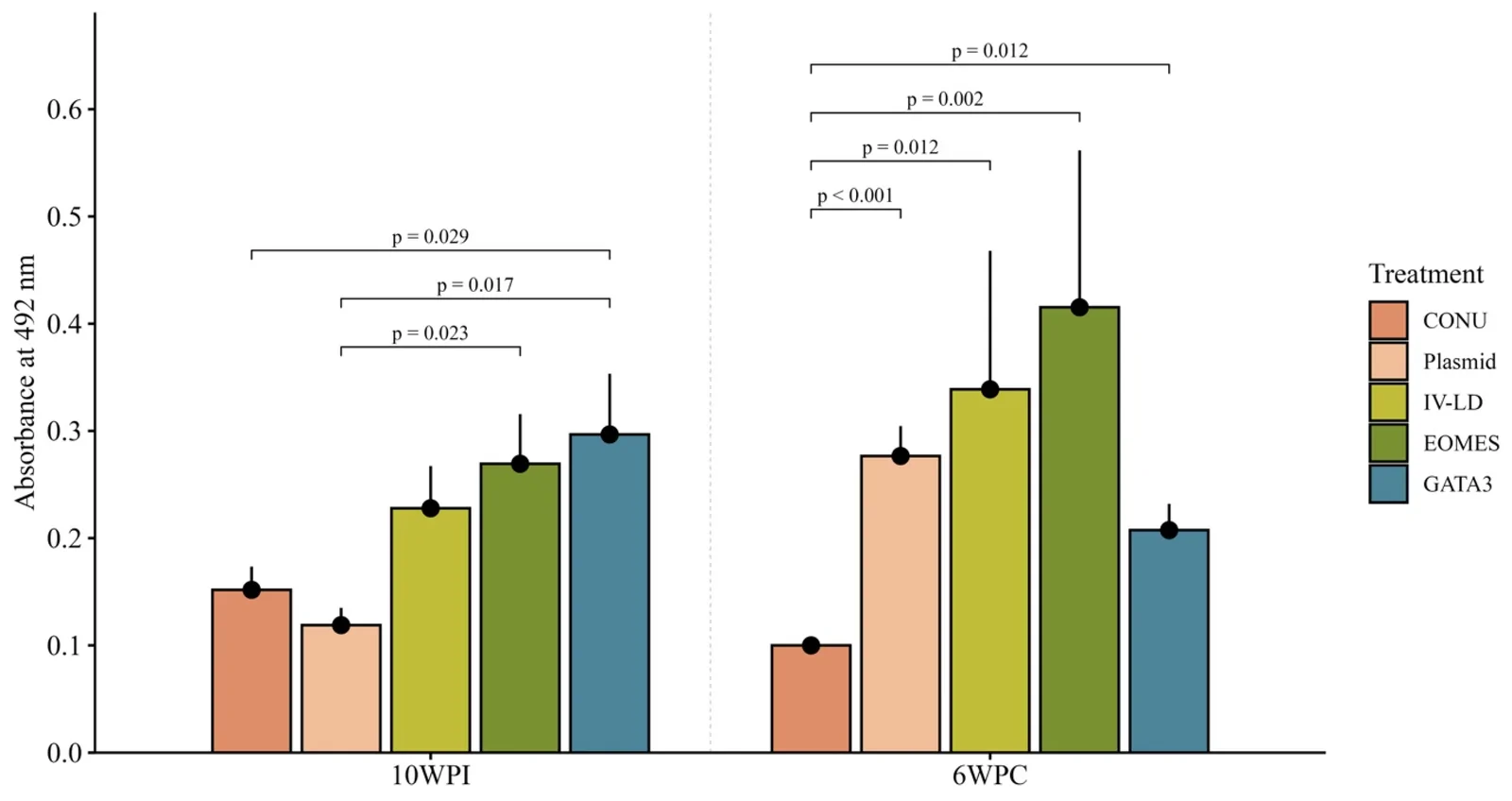
Enzyme-linked immunosorbent assay (ELISA) results measuring antibody levels at 10 weeks post-immunization (left) and 6 weeks post-challenge (right). Bars represent mean absorbance at 492 nm ± standard error of the mean (SEM) for fish receiving one of three vaccines (IV-LD, EOMES, GATA3), a plasmid-only control (Plasmid), or a non-immunized control (CONU); sample sizes (n) are indicated below the bars. Statistical differences among treatment groups were evaluated using the Kruskal–Wallis non-parametric test, followed by *Dunn’s* post hoc test with *Benjamini–Hochberg* correction for multiple comparisons. *Adjusted p-values* are shown above significant pairwise comparisons.

At 10 weeks post-immunization, fish vaccinated with the adjuvants had significantly higher antibody levels when compared to fish of the Plasmid group, with peak absorbance values reaching 0.27 for EOMES (*adjusted p-value =* 0.023) and 0.3 for GATA3 (*adjusted p-value =* 0.017). The difference in absorbance at 492 nm was also significantly higher in GATA3 compared to CONU (*adjusted p-value =* 0.029). Although the low dose of inactivated virus (IV-LD) appeared to show a higher antibody response compared to the non-immunized (CONU) and Plasmid (non-coding) control groups, this difference was not statistically significant (Figure 4).

By 6 weeks post-challenge, the groups that were injected with IV-LD + *gata3*, IV-LD + *eomes*, IV-LD alone or control non-coding plasmid exhibited significantly (*adjusted p-values* = 0.012, 0.002, 0.012, <0.001, respectively) higher antibody levels compared to the saline control group. Mean antibody levels appeared to have risen in Plasmid, IV-LD, and EOMES groups, while a slight decline was observed in the GATA3 group. However, only the Plasmid group showed a statistically significant difference in antibody levels between timepoints (*Wilcoxon rank-sum* test with *Benjamini–Hochberg* correction, *adjusted p-value = 0.0024*). A considerable degree of variability was observed within the IV-LD and EOMES groups, indicating that some individuals mounted a more robust antibody response following the SAV3 challenge.

### 3.4. Virus Neutralization Activity of Serum Samples

**Figure 5:**
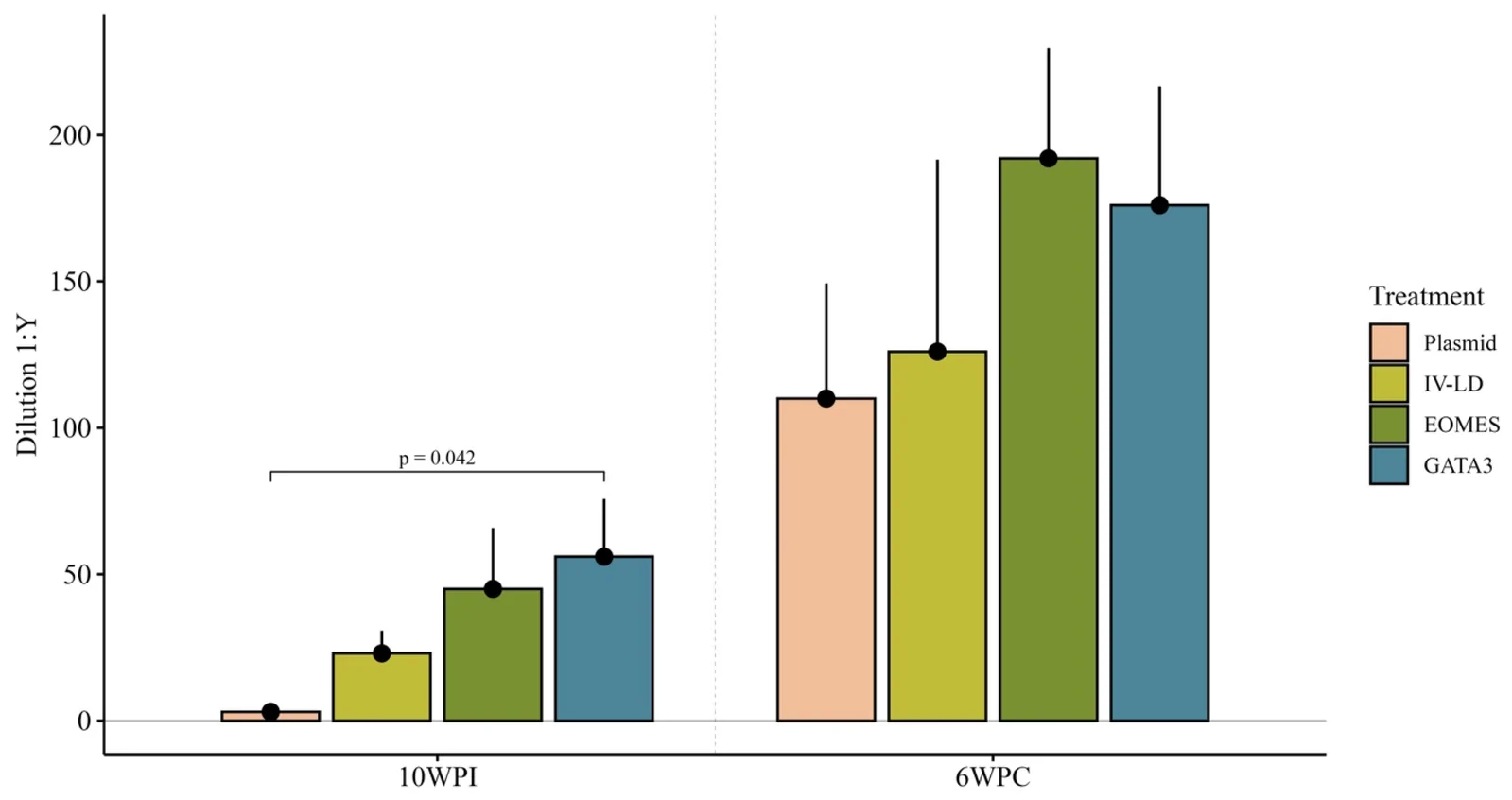
Results of the virus neutralization assay assessing the ability of serum from immunized Atlantic salmon to inhibit SAV3-induced cytopathic effects (cell lysis or necrosis). Neutralizing antibody titers (expressed as the reciprocal of the highest serum dilution showing protection) were assessed at 10 weeks post-immunization (left) and 6 weeks post-challenge (right). Bars represent mean neutralizing antibody titers ± standard error of the mean (SEM) for fish receiving one of three vaccines (IV-LD, EOMES, GATA3), or a plasmid-only control (Plasmid). Statistical differences among treatment groups were evaluated using the Kruskal–Wallis non-parametric test, followed by *Dunn’s* post hoc test with *Benjamini–Hochberg* correction for multiple comparisons. *Adjusted p-values* are shown above significant pairwise comparisons.

In general, the levels of virus neutralization activity possessed by the serum samples from Atlantic salmon in all groups resembled the ELISA results—relatively high virus neutralization activity in serum samples from fish that received the molecular adjuvants, and lower virus neutralization activity in the serum of the Plasmid and IV-LD, at 10WPI and 6WPC. Serum samples from fish that received saline were not included in this assay. The only statistical difference encountered within a timepoint was between GATA3 and Plasmid at 10WPI (*adjusted p-value* = 0.042). Compared to the plasmid control, serum samples from all vaccine groups demonstrated a higher virus neutralizing activity at 10 weeks post-immunization (Figure 5). While serum from the Plasmid control group required minimal dilution to neutralize SAV3 infection, serum from fish immunized with IV-LD combined with *eomes*- or *gata3-* encoding plasmids effectively neutralized the virus at dilutions of up to 45× and 56×, respectively. The virus neutralization activity in sera from fish that were given non-coding plasmid DNA was somewhat surprising—but follow the pattern seen from antibody response.

A significant difference in virus neutralization capability was observed between timepoints before and after challenge (*Wilcoxon* rank-sum test, *p =* 2.82 × 10^−8^). This was reflected in an overall increase in serum dilution required to block SAV3 infection in CHH-1 cells across all treatment groups. The lowest neutralizing titer post-challenge was observed in the plasmid control group (1:110), while the highest titer (1:192) was detected in fish that received IV-LD combined with an EOMES-encoding plasmid. These results indicate that serum from all vaccinated groups retained the ability to inhibit viral infection following challenge.

### 3.5. Viral Load

**Figure 6:**
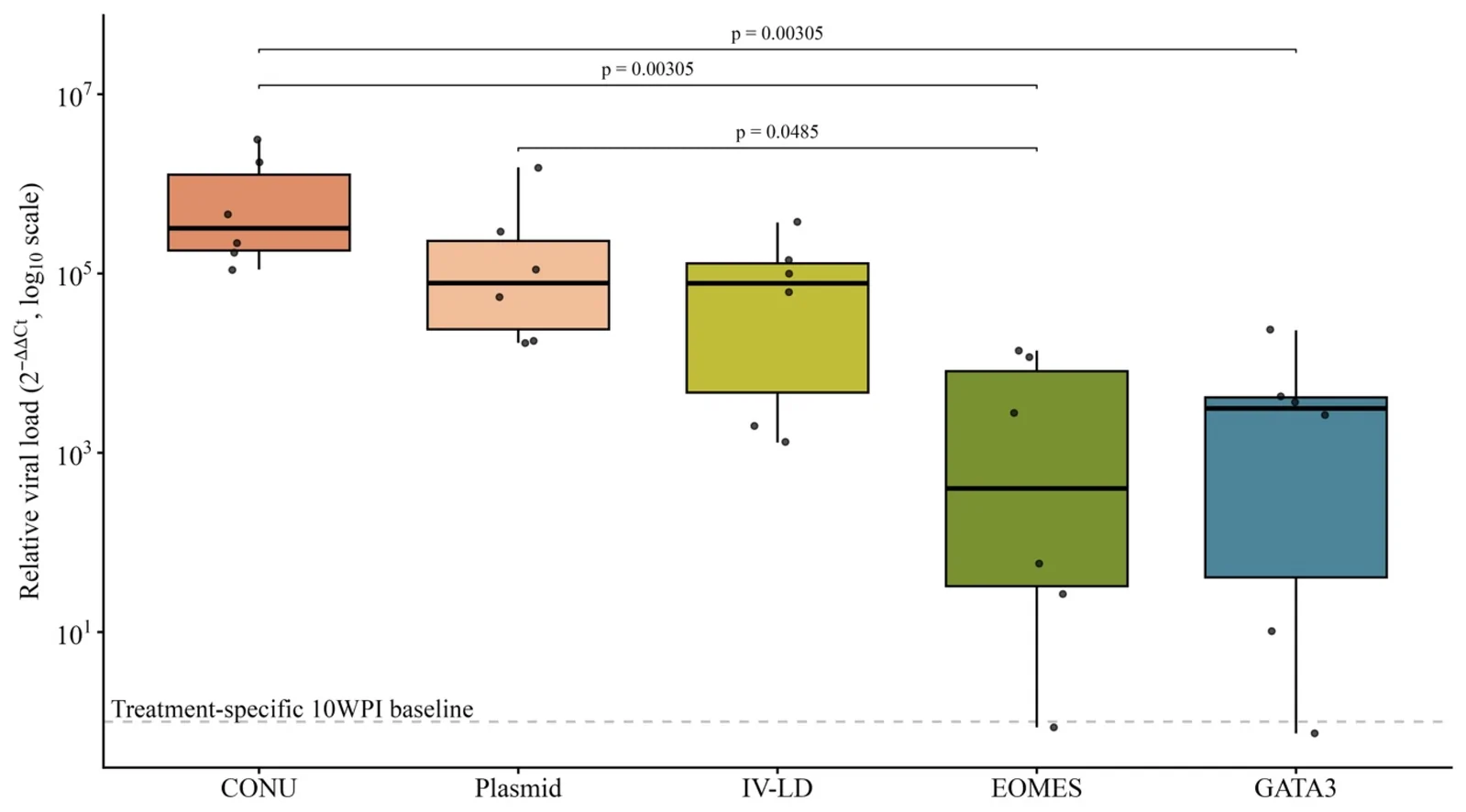
Relative viral load in Atlantic salmon following challenge with SAV3. Viral load was quantified in heart tissue by RT-qPCR at 4 weeks post-challenge, expressed as fold-change relative to the mean 10 weeks post-immunization baseline of the corresponding treatment group using the $2^{-\Delta \Delta Ct}$ method (log_10_ scale). Boxplots show median, interquartile range, and individual fish values for each treatment group: non-immunized control (CONU), plasmid-only control (Plasmid), IV-LD, EOMES, and GATA3. Statistical differences between treatment groups were evaluated using the Kruskal–Wallis non-parametric test, followed by *Dunn’s* post hoc test with *Benjamini–Hochberg* correction for multiple comparisons. *Adjusted p-values* are shown above significant pairwise comparisons.

Viral load in the heart at 4 WPC was quantified as relative fold-change ($2^{-\Delta \Delta Ct}$) using 10 WPI as the baseline reference (Figure 6). The relative virus load in the GATA3 and EOMES group was significantly lower than in the control group (*Dunn’s* test, *adjusted p-value* = 0.00305), whereas fish in the EOMES group contained significantly less virus than fish belonging to the Plasmid group (*Dunn’s* test, *adjusted p-value* = 0.0485). There were no significant statistical differences between the other vaccine groups.

### 3.6. Histopathology Scoring (Histoscoring)

The extent of the lesions in the pancreas and heart of Atlantic salmon caused by SAV3 were evaluated at 4 weeks post-challenge (Supplementary Figures S1 and S2). Although the heart is one of the primary reservoirs for SAV3 in Atlantic salmon and usually exhibits both inflammation (primarily epicardial) and myocyte necrosis (primarily myocardial), the pancreas is also affected by the virus, primarily through acinar cell necrosis. Pancreatic necrosis is often apoptotic in nature and may occur with minimal to no inflammation. When pancreatic inflammation is observed in trials, it may be attributed to local effects of vaccine injection rather than direct viral pathology.

**Figure 7:**
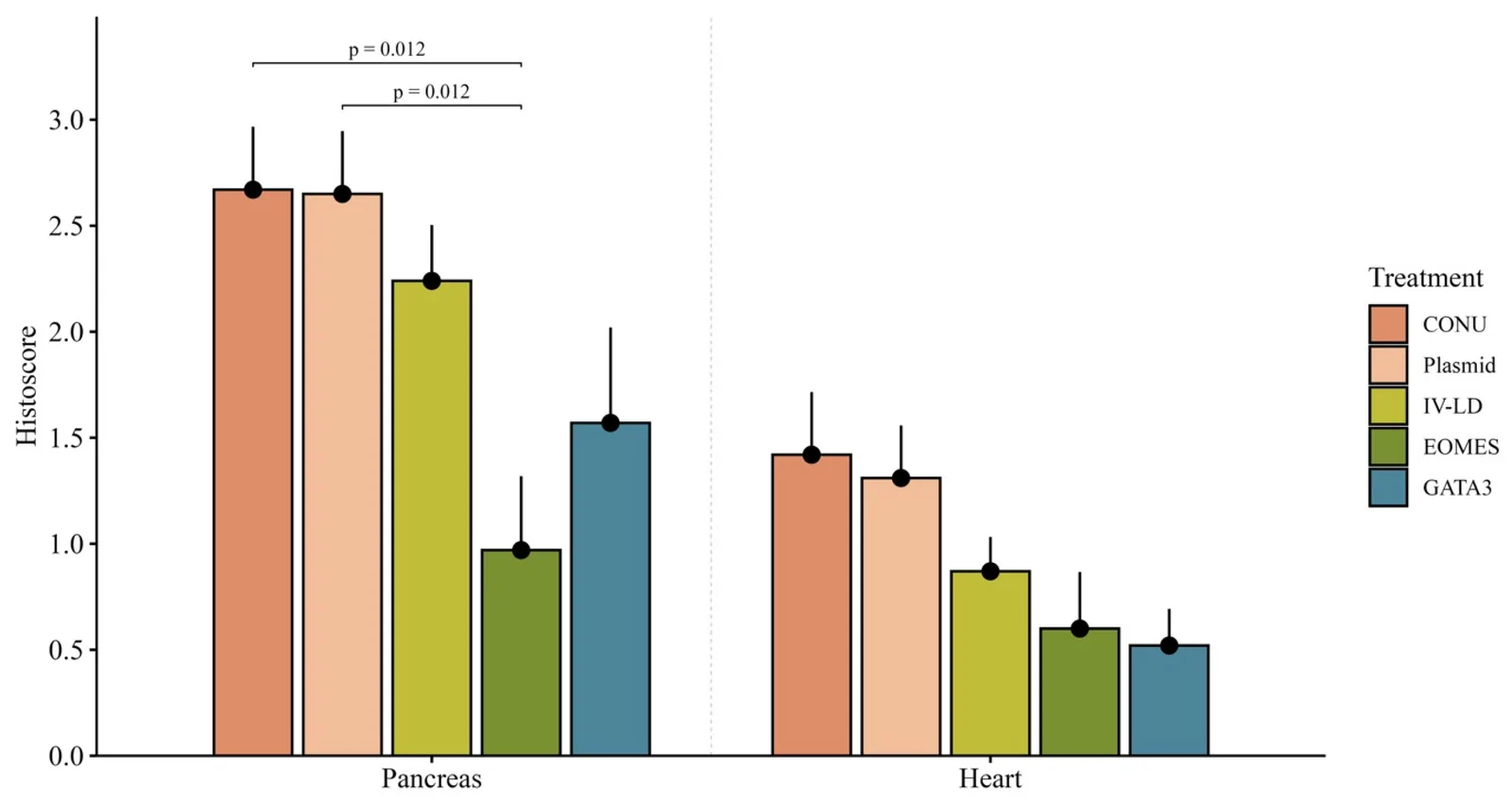
Histopathological scoring (histoscore) of the pancreas (left) and heart (right) tissues at 4 weeks post-challenge. Bars represent mean histoscore ± standard error of the mean (SEM), reflecting tissue pathology, for fish treated with one of three vaccines (IV-LD, EOMES, GATA3), a plasmid-only control (Plasmid), or a non-immunized control (CONU). Statistical differences between treatment groups were evaluated using the Kruskal–Wallis non-parametric test, followed by *Dunn’s* post hoc test with *Benjamini–Hochberg* correction for multiple comparisons. *Adjusted p-values* are shown above significant pairwise comparisons.

At 4 WPC, vaccination with EOMES was associated with a significant decrease in pancreas histoscore, representing a relative reduction in pancreatic damage when compared to either the CONU (*adjusted p-value =* 0.02) or Plasmid (*adjusted p-value =* 0.0218) groups. There were no other significant differences in pancreas histoscores, although overall scores were lowest in the three vaccinated groups (Figure 7).

**Figure 8:**
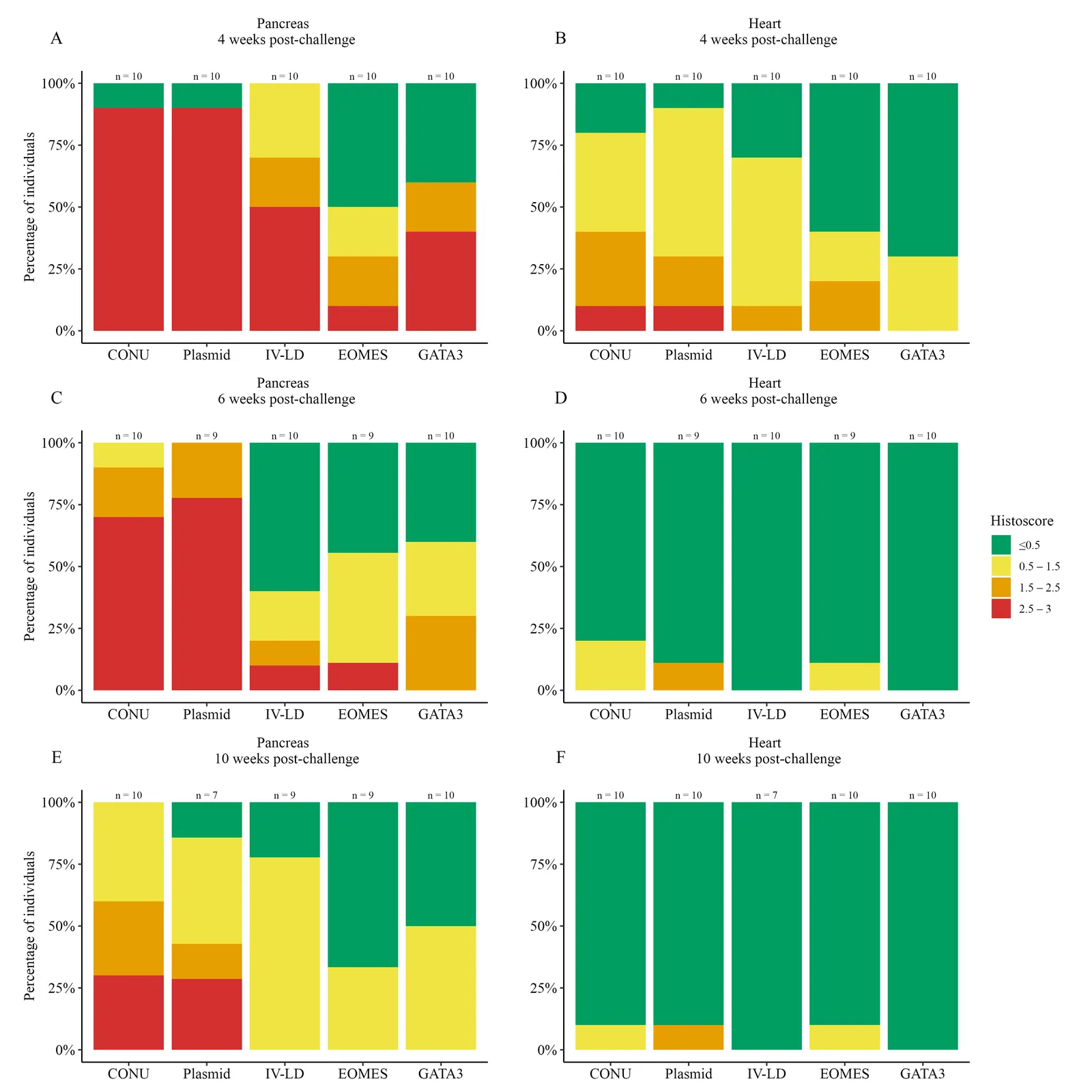
Histoscore distributions in the pancreas (left panels: (**A**,**C**,**E**)) and heart (right panels: (**B**,**D**,**F**)) tissues of Atlantic salmon at 4, 6, and 10 weeks post-challenge. Each panel compares the effects of three vaccine treatments (IV-LD, EOMES, GATA3), a plasmid control (Plasmid), and a non-immunized control (CONU). Bars represent the percentage of individuals falling into four histoscore categories: 0.5 (green); 0.5–1.5 (yellow); 1.5–2.5 (orange); 2.5–3 (red). Histoscore reflects the severity of inflammation/damage based on histopathological scoring. Numbers above each bar indicate sample size (n).

The semi-quantitative assessment-based histopathology scoring showed that for CONU and Plasmid, most individuals had severe damage to the pancreatic tissue and IV-LD fish had moderate to severe, while approximately 50% of the fish from the molecularly adjuvanted vaccine groups had few heart lesions (Figure 8A)—which can be translated into a proxy for increased vaccine efficacy. Looking at mean histoscore levels in Figure 6, the fish that received IV-LD together with *eomes*-encoding plasmid performed significantly better, compared to CONU (*adjusted p-value = 0.002*) and Plasmid (*adjusted p*-value *= 0.002*) with respect to vaccine efficacy. In the heart, at 4 WPC, over 75% of individuals in the control groups exhibited moderate to severe lesions (Figure 8B; histoscore 0.5–3). Over 50% of the individuals in EOMES and GATA3 had healthy heart tissue, with low histoscore (Figure 8B).

At 6 and 10 weeks post-challenge, most of the hearts had recovered based on histoscore results (Figure 8D,F). At week 6 post-challenge, the fish that were vaccinated with control plasmid and saline showed the highest histoscores while IV-LD + EOMES and IV-LD + GATA3 showed less pathology. Ten weeks after experimental challenge, the fish that were injected with saline and those vaccinated with the non-coding plasmid still showed pathology in the pancreas, whereas the fish vaccinated with IV-LD, IV-LD + EOMES and IV-LD + GATA3 performed the best. A similar tendency was observed in heart tissue at 6 and 10 WPC, with the difference that most fish in all groups had a histoscore lower than 0.5.

Collectively, the vaccines comprising IV-LD in combination with molecular adjuvants (EOMES or GATA3) conferred superior protection when compared to IV-LD alone or the non-coding plasmid, indicating that the adjuvanted formulations elicited a higher level of protection against SAV3-induced lesions.

## 4. Discussion and Conclusions

This study demonstrates that *gata3* and *eomes* as molecular adjuvants have the potential to enhance the performance of an inactivated SAV3 vaccine in Atlantic salmon. Our findings show that including plasmids encoding the *gata3* and *eomes* transcription factors, as molecular adjuvants to vaccine, led to increased antibody production, reduced pancreas lesions, and improved growth performance compared to non-adjuvanted vaccine and controls. This highlights the potential of these molecular adjuvants in enabling robust, targeted immunity against SAV3 in Atlantic salmon.

The current study revealed transgenic expression of both *eomes* and *gata3* both in vitro and in vivo. This is in accordance with other work on Atlantic cod (*Gadus morhua*) [54], rainbow trout (*Onchorhynchus mykiss*) [55] and A. salmon [33]. From the in vitro study, the expression of *gata3* and *eomes* was confined to the cell nuclei, which indicates that they may be able to bind DNA, potentially inducing a response. The presence of transgenes within the connective tissue cells, fibroblast-like cells or leukocytes associated with the fascial layer surrounding muscle cells has not been previously demonstrated. Such fibroblasts produce connective tissue substances forming the endomysial fascia [56], but fibroblasts are indeed involved in several immune pathways such as monocyte, lymphocyte, eosinophil and neutrophil recruitment, dendritic cell (DC) activation, antiviral activity by secretion of a variety of cytokines and other immune factors that may be able to induce immune responses. A key challenge with plasmid-based vaccines is the rapid clearance of plasmid DNA (pDNA) from tissues and its subsequent degradation, leading to reduced pDNA uptake and limited transgene expression [57]. This has been observed before in Atlantic salmon, [58], that circularized DNA constructs can be expressed in tissues away from the site of injection. A study on Atlantic salmon found expressions of *gata3* and Tbet in leucocytic cells in the inflammatory foci—at the site where the plasmid DNA were injected [33].

### 4.1. Antibody Response

Inactivated virus particles are normally poor immunogens giving low antibody response and, thus, requiring high or repeated doses [59]. This was also the case in our study where the inactivated low dose SAV vaccine gave poor antibody response. However, when the IV-LD SAV3 virus particle vaccine was adjuvanted with *eomes* and *gata3*-encoding plasmids, the resulting antibody response was higher than in fish that received saline and inactivated low dose virus. Of note, the antibody response, post-SAV challenge, in fish that got naked control plasmid was also substantial, indicating that naked plasmids also possess adjuvant activity. This phenomenon is acknowledged since backbone plasmid DNA may contain immunostimulatory CpG motifs which may bind to TLR9 [60]. In the current study, the relationship concerning whether TLR9 affected antibody response is not known. In one study in mice, certain TLR9 ligands (CpGs) were found to increase antibody response but failed to promote affinity maturation [61]. A study in salmon partly confirmed that CpG induces elevated antibody response (head kidney and splenic IgM^+^ cells). None of these studies used naked plasmid DNA [62].

Molecular adjuvants, as co-injected with inactivated pathogens, have shown some degree of success. While most of these were pattern recognition receptor (PRR) ligands, nowadays cytokines are seen as a very promising adjuvant [25]. In this study, we show that transcription factors, *eomes* and *gata3,* can be a good adjuvant candidate and possess the ability to elicit a higher antibody response than fish injected with a control plasmid or a low dose of inactivated virus vaccine.

Gata3 is recognized as a master regulator of innate and adaptive immunity, crucial for modulating various immune cells and cytokines [63], and is the only member of the GATA family of transcription factors that is expressed in several cell types. Gata3 aids in the differentiation of naïve T cells to CD4+ T helper 2 cells, contributing to cell-mediated immunity against intracellular and extracellular pathogens. Effector Th2 cells are pivotal in activating B lymphocytes by releasing cytokines that drive B cell proliferation and differentiation into antibody-secreting plasma cells [64]. The initial differentiation of naïve T cells into effector Th2 cells is triggered by antigen binding to the T-cell receptor (TCR), while the presence of gata3 amplifies its expression through a positive feedback mechanism, further promoting Th2 differentiation [65,66]. Despite most Th2 cells undergoing apoptosis after antigen clearance, some of the effector cells transition into memory cells, providing the immune system with a quick response resource in case of re-infection. Although eomes is predominantly associated with the promotion of cytotoxic T cell responses and the activation of natural killer (NK) cells, it is also playing a role in T helper 1 cell-mediated immune responses [67]. One theory behind a stronger antibody response is that Gata3, together with Stat5, controls the differentiation of naïve T cells to Th2 cells, which produce cytokines such as IL-4. IL-4 may, in turn, increase gata3 expression, thereby sustaining Th2 polarization [68]. Whether this simplified theory is true in the current study is not clear. On the other hand, eomes may compete with Gata3 by competitive binding to the IL5 promoter. In this case, one may expect a lower IL5 production and antibody response. This was not the case in our study—as there was no significant difference in antibody response between fish that received *gata3*- or *eomes*-containing plasmid adjuvants. However, there was a clear difference between antibody response in fish, at 6 WPC, that received the control plasmid and the plasmid encoding Gata3. The reason for this is not known. One may speculate that other regulatory proteins or/and transcription factors may interfere with Gata3. Possible factors that may regulate Gata3 activity are Sox4, TGFß, Fog1 and Rog [69,70,71]. An approach involving, e.g., a gene expression of these regulatory factors may give more knowledge as to what happened in the current study.

### 4.2. Virus Neutralization

Virus neutralization is defined as the reduction in viral infectivity mediated by antibodies, both vaccine-induced and natural antibodies (neutralizing antibodies [NAbs]) [72]. By binding to the virions, NAbs block one or more steps of the viral replication cycle (i.e., entry, binding to cellular receptors, uncoating), and/or cause aggregation or lysis of virus particles, thus neutralizing or reducing virus infectivity [73]. NAbs may be considered a correlate of protection from viral infection after vaccination. These findings may account for the minor discrepancies observed between antibody concentration and virus neutralization capability at 6 weeks post-challenge. However, there is a general consistency between the ELISA and VNA results, aside from the lower antibody levels for the GATA3 group compared to the other groups at 6 weeks post-challenge.

### 4.3. Histopathology and Vaccine Efficacy

The SAV3 has a tropism for heart and pancreas tissue, where the tissues suffer from inflammation during virus infection—causing lesions [74]. Usually, vaccine efficacy is evaluated by the percentage of survival after an experimental challenge with a homologous pathogen [75]. Experimental challenge with salmonid alphavirus (SAV) will, in general, either not cause any post-challenge mortality, or very low mortality (10–12%). The key changes following experimental or natural challenge are necrosis/loss of the exocrine pancreas and various degrees of heart lesions (individual cell necrosis and inflammation), which are consistent across SAV genotypes. Vaccines (or prophylactic measures) are experimentally tested for their ability to reduce pancreas and heart scores [76]. The effect on growth and growth arrest can be found here: [77]. On this basis, assessment of the impact on pathological changes in the pancreas and heart constitutes a good correlate of what is found under field conditions and validates the method used.

In this study, there was no mortality, meaning that it is reasonable to assess regeneration of pancreas and/or heart as a proxy for vaccine efficacy or performance. In the current study, the histoscores were lower at 4 weeks post-challenge, in hearts and pancreas from fish vaccinated with IV-LD, IV-LD + *gata3*-encoding plasmid, and IV-LD + *eomes*-encoding plasmid. Four weeks post-challenge has previously been shown to be an optimal timepoint to assess the heart lesions or regeneration, based on the infection biology of SAV3 [78]. In the current study, the histoscore parallels a previous report that an inactivated SAV3 vaccine lowers the heart lesions [79] while there are no reports how vaccines with molecular adjuvants, such as *gata3* and *eomes*, perform in a SAV3-vaccine performance study.

### 4.4. Limitations of the Study

As the functional tools (e.g., antibodies and specific inhibitors for different T cell differentiation stages for use in flow cytometry) for studying distinct molecular T cell pathways are lacking for Atlantic salmon, we cannot depict specific pathways and molecular response that give disease protection against pancreas disease. Gene editing to delete any T cell pathways in Atlantic salmon requires several years and as yet no 100% successful knock-out salmon has been developed, in contrast to zebrafish with a quick reproduction rate. We have undertaken rigorous RNA sequencing of several tissues 10 weeks post immunization and during SAV infection, to look for any gene signature(s) that may be a correlate of protection—or any T-cell pathways that were significantly regulated. This effort did not yield any conclusive results. We may speculate that very early gene signatures are decisive for T-cell differentiation and we missed the specific early timepoint. Future work should exploit knowledge from innate immune response(s) and gene signatures therein to develop a precision vaccinology platform tailoring hard-to-combat pathogens. We believe that early response may be decisive for which adaptive response happens. However, using the standard vaccine response assays in the present study, we believe that antibody response and virus neutralization are vital for protection. We could not detect any regulation of *gata3* and *eomes* mRNA during the immunization phase, probably due to a too late sampling timepoint. The histoscoring was performed by an experienced fish pathologist on coded samples. The assessment was later confirmed by a fish health candidate (equivalent to a veterinarian but specialized in fish) with an overall agreement with the pathologist. We think the pathology assessment is robust, but we also see that there could be a third, experienced, pathologist involved.

In conclusion, it was demonstrated that the injection of plasmids encoding *gata3* and *eomes* together with a vaccine consisting of inactivated SAV3 induced a high specific antibody response and increased the vaccine performance compared to inactivated virus vaccine. However, naked non-coding plasmid DNA, used as control, also exhibited a certain stimulatory effect on antibody response. The use of T cell-derived molecular adjuvants encoded in plasmid DNA opens new opportunities for the use of immune pathway-specific adjuvants in precision vaccinology.

## Figures and Tables

**Table 1:** Semi-quantitative histoscoring based on [40]. Tissue degeneration and inflammation in pancreas (P) and heart (H) were evaluated and graded. See Supplementary Figures S1 and S2 for typical histoscore examples (pancreas and heart).

Grade	Heart	Pancreas
0	Normal structure	Normal structure
1 (Mild)	Multifocal myocarditic necrosis and/or inflammation (<15% of fibers affected)	Focal acinar cell necrosis (<15% of fibers affected)
2 (Moderate)	Multifocal myocarditic necrosis and/or inflammation (15–50% of fibers affected)	Significant multifocal necrosis/atrophy of pancreatic acinar tissue (15–50% of fibers affected)
3 (Extensive)	Extensive diffuse myocarditic necrosis and/or inflammation (>50% of fibers affected)	Total absence of acinar cells and tissue (>50% of fibers affected)

## Data Availability

Please contact corresponding authors for data. Data analysis scripts can be found on https://github.com/UiT-RGG/figueiredo-et-al_2026 (1 June 2026).

## Supplementary Materials

The following supporting information can be downloaded at: https://www.mdpi.com/article/10.3390/pharmaceutics18081033/s1, Table S1: Primers and primer sequences used in this study, with GenBank accession or catalogue number; Table S2: Vaccine groups (and designations), vaccine type, and doses used in the immunisation trial; Table S3: Pairwise effect sizes (*Cohen’s d*) for specific growth rate (SGR) comparisons between treatment groups at relevant timepoints; Figure S1: Shows pancreas with different pathology levels; Figure S2: Shows heart with different pathology levels.
