# Supplementary material for: Plasmid-DNA Encoded gata3 and eomes Increase Antibody Response and Performance of an Inactivated Salmonid Alphavirus Subtype 3 Vaccine
Source: Pharmaceutics. 2026 Aug 20;18(8):1033. doi: 10.3390/pharmaceutics18081033 (PMC13516754; doi:10.3390/pharmaceutics18081033)
Supplement: Supplementary file 1 [file pharmaceutics-18-01033-s001.zip › pharmaceutics-4452377-supplementary.pdf]

SUPPLEMENTARY MATERIALS

Supplementary Table S1. Primers and primer sequences used in this study, with GenBank accession or catalogue number

Primer	Sequence	GenBank/Catalogue number
GATA-3 forward	TCAGATCTCGAGATGGAAGTATCCGCCGACCAACCCC	EU418015.1
GATA-3 reverse	CCGGGCCCGCGGGCCCATGGCAGAGACCATACTG	
Eomes forward	TCAGATCTCGAGATGCAGCTGGAGAATATTCTTCC	EU418014.1
Eomes reverse	CGTCGACTGCAGGGGGCTTGCGTAAAAGGCATAG	
pTagRFP-N forward	ACAACTCCGCCCATTGACGCAAAT	#FP142
pTagRFP-N reverse	CCGCCCTCGACCACCTTGATTCTCATG	

Supplementary Table S2: Vaccine groups (and designations), vaccine type, and doses used in the immunisation trial

Vaccine	Type	Dose
CONU - unvaccinated control	Saline	0.1 ml
p-TagRFP (empty plasmid)	pDNA	1 μg g^{-1} fish
Low dose inactivated virus (IV-LD)	Oil-adjuvanted	10^9 $TCID_{50}$
Low dose inactivated virus + *gata3*-encoding plasmid (GATA3)	Oil-adjuvanted + pDNA	10^9 $TCID_{50}$ + 1 μg g^{-1} fish
Low dose inactivated virus + *eomesodermin*-encoding plasmid (EOMES)	Oil-adjuvanted+ pDNA	10^9 $TCID_{50}$ + 1 μg g^{-1} fish

Supplementary Table S3: Pairwise effect sizes (*Cohen's d*) for specific growth rate (SGR) comparisons between treatment groups at relevant timepoints. Effect sizes were calculated between all combinations of treatment groups within each sampling period: Immunisation - 10 weeks post-immunisation (10WPI), Challenge - 4 weeks post-challenge (4WPC), and Challenge - 10 weeks post-challenge (10WPC). Positive values indicate higher SGR in the second group of the pair, while negative values indicate higher SGR in the first group. Effect sizes were interpreted following conventional benchmarks: **negligible** (|d| < 0.2), **small** (0.2 ≤ |d| ≥ 0.5), **medium** (0.5 ≤ |d| ≥ 0.8), and **large** (|d| ≥ 0.8). These standardize differences complement the *adjusted p-value* based analyses by quantifying the magnitude and practical relevance of between-group differences.

Period	Group 1	Group 2	Effect size	Interpretation
Immunisation - 10WPI	CONU	IV-LD	-0.11	negligible
Immunisation - 10WPI	CONU	EOMES	-0.47	small
Immunisation - 10WPI	CONU	GATA3	-0.26	small
Immunisation - 10WPI	CONU	Plasmid	-1.28	large
Immunisation - 10WPI	IV-LD	EOMES	-0.36	small
Immunisation - 10WPI	IV-LD	GATA3	-0.14	negligible
Immunisation - 10WPI	IV-LD	Plasmid	1.23	large

Immunisation - 10WPI	EOMES	GATA3	0.24	small
Immunisation - 10WPI	EOMES	Plasmid	1.04	large
Immunisation - 10WPI	GATA3	Plasmid	1.18	large
Challenge - 4WPC	CONU	IV-LD	-1.46	large
Challenge - 4WPC	CONU	EOMES	-1.64	large
Challenge - 4WPC	CONU	GATA3	-1.08	large
Challenge - 4WPC	CONU	Plasmid	-0.46	small
Challenge - 4WPC	IV-LD	EOMES	0.04	negligible
Challenge - 4WPC	IV-LD	GATA3	0.19	negligible
Challenge - 4WPC	IV-LD	Plasmid	-0.73	medium
Challenge - 4WPC	EOMES	GATA3	0.18	negligible
Challenge - 4WPC	EOMES	Plasmid	-0.76	medium
Challenge - 4WPC	GATA3	Plasmid	-0.50	medium
Challenge - 10WPC	CONU	IV-LD	-0.94	large
Challenge - 10WPC	CONU	EOMES	-1.21	large
Challenge - 10WPC	CONU	GATA3	-0.99	large
Challenge - 10WPC	CONU	Plasmid	0.29	small
Challenge - 10WPC	IV-LD	EOMES	-0.39	small
Challenge - 10WPC	IV-LD	GATA3	-0.25	small
Challenge - 10WPC	IV-LD	Plasmid	-1.14	large
Challenge - 10WPC	EOMES	GATA3	0.04	negligible
Challenge - 10WPC	EOMES	Plasmid	-1.37	large
Challenge - 10WPC	GATA3	Plasmid	-1.18	large

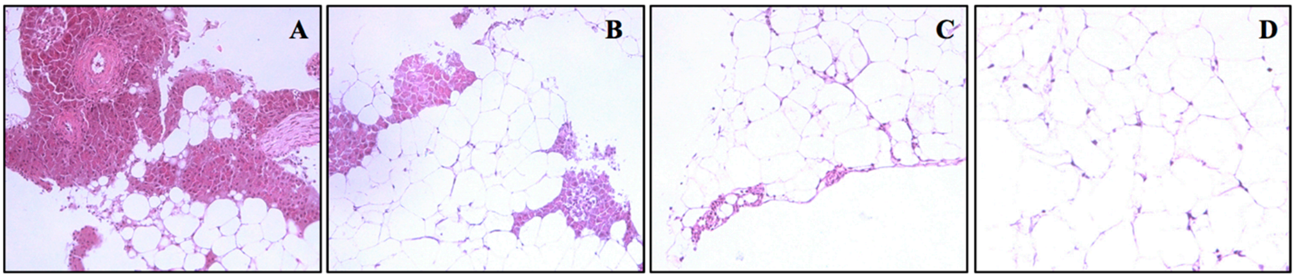

Supplementary Figure S1: Shows pancreas with different pathology levels. A) Grade 0; B) Grade 1; C) Grade 2 and D) Grade 3. For explanation, see table 1

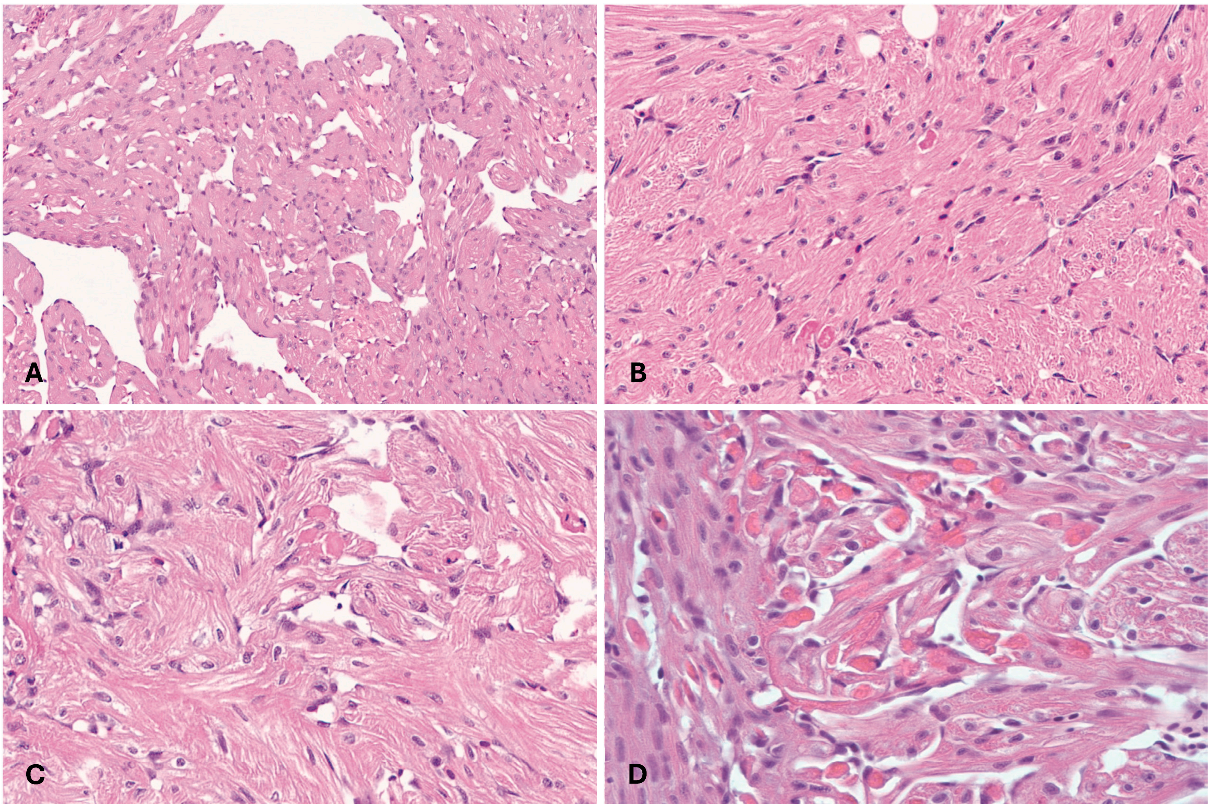

Supplementary Figure S2: Shows heart with different pathology levels. A) Grade 0; B) Grade 1; C) Grade 2 and D) Grade 3. For explanation, see table 1.
